# Supplementary material for: Chronic Intermittent Hypoxia Exposure Alternative to Exercise Alleviates High-Fat-Diet-Induced Obesity and Fatty Liver
Source: Int J Mol Sci. 2022 May 6;23(9):5209. doi: 10.3390/ijms23095209 (PMC9104027; doi:10.3390/ijms23095209)
Supplement: Supplementary file 1 [file ijms-23-05209-s001.zip › ijms-1664714-supplementary.pdf]

Supplemental material

Figure S1. The H&E staining of heart, lung and kidney tissues.

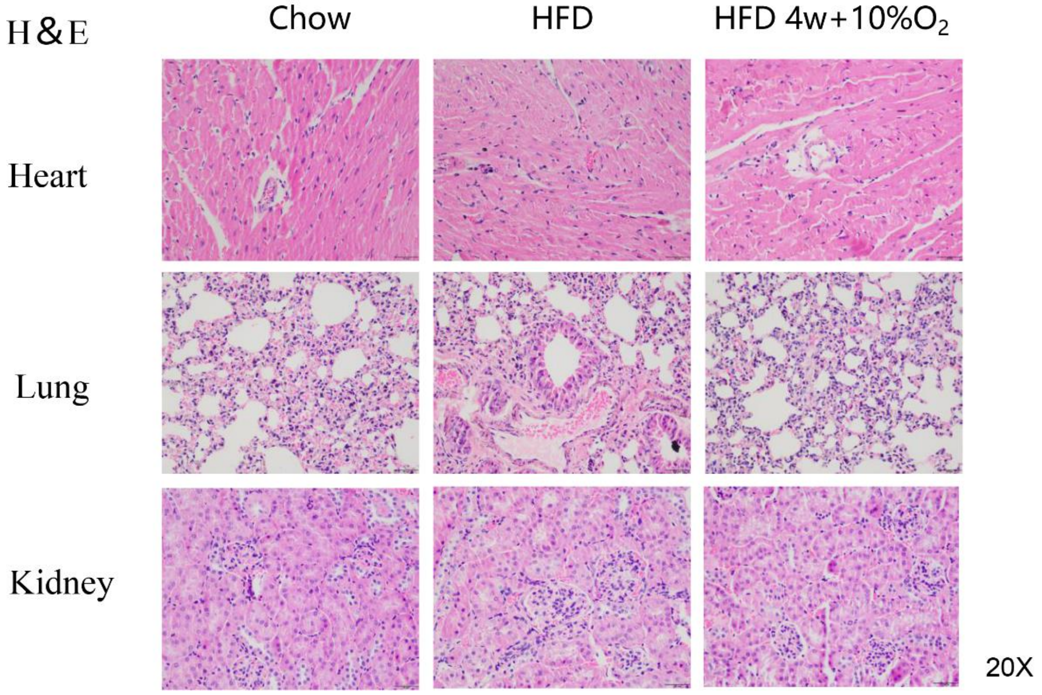

Table S1. *RT-PCR* primers

18S rRNA-Forward	5'-GCAATTATTCCCCATGAACG -3'
18S rRNA-Reverse	5'-GGCCTCACTAAACCATCCAA-3'
SCD1-Forward	5'-TTCTTGCGATACACTCTGGTGC-3'
SCD1-Reverse	5'-CGGGATTGAATGTTCTTGTCGT-3'
Cpt1a-Forward	5'-CTCCGCCTGAGCCATGAAG-3'
Cpt1a-Reverse	5'-CACCAGTGATGATGCCATTCT-3'
PGC1α-Forward	5'-TATGGAGTGACATAGAGTGTGCT-3'
PGC1α-Reverse	5'-CCACTTCAATCCACCCAGAAAG-3'
Atgl-Forward	5'-GGATGGCGGCATTTCAGACA-3'
Atgl-Reverse	5'-CAAAGGGTTGGGTTGGTTCAG-3'
PPARα-Forward	5'-TTCTTGCGATACACTCTGGTGC-3'
PPARα-Reverse	5'-CGACAGACAGGCACTTGTGAAA-3'
UCP1-Forward	5'-AGGCTTCCAGTACCATTAGGT-3'
UCP1-Reverse	5'- CTGAGTGAGGCAAAGCTGATTT-3'
ADR3-Forward	5'- ACTTGGTAGTGGGACTCCTCG T -3'
ADR3-Reverse	5'- TGCTGGCAGTTACAC AGAGC -3'
arginase-Forward	5'- CTCCAAGCCAAAGTCCTTAGAG -3'

arginase -Reverse	5'- AGGAGCTGTCATTAGGGACATC -3'
CD206 -Forward	5'- TGGAAGAAGAAGTAGCCTATC -3'
CD206 -Reverse	5'- TGGAGTAGTGGTTGGAGAA -3'
UCP2--Forward	5'- ATGGTTGGTTTCAAGGCCACA-3'
UCP2--Reverse	5'-CGGTATCCAGAGGGAAAGTGAT-3'
adiponectin-Forward	5'-TGTTCCTCTTAATCCTGCCCA-3'
adiponectin-Reverse	5'-CCAACCTGCACAAGTTCCCTT-3'
C/EBP-Forward	5'-CAAGAACAGCAACGAGTACCG-3'
C/EBP-Reverse	5'-GTCACTGGTCAACTCCAGCAC-3'
